# Maternal health education and social support needs across the perinatal continuum of care: a thematic analysis of interviews with postpartum women in Punjab, India

**DOI:** 10.1186/s12884-025-07813-8

**Published:** 2025-07-02

**Authors:** Preetika Sharma, Rashmi Bagga, Maliha Khan, Mona Duggal, Darshan Hosapatna Basavarajappa, Alka Ahuja, Ankita Kankaria, Nadia Diamond-Smith, Vijay Kumar, Manju Kashyap, Pushpendra Singh, Jasmeet Kaur, Alison M. El Ayadi

**Affiliations:** 1 Postgraduate Institute of Medical Education & Research, Chandigarh, India; 2 University of California, San Francisco, San Francisco, USA; 3 All India Institute of Medical Sciences, Bathinda, India; 4 Survival of Women and Children Foundation, Panchkula, India; 5 Indraprastha Institute of Information Technology Delhi, New Delhi, India

**Keywords:** Social support, Health education, Pregnancy, Childbirth, Postpartum, Postnatal, India

## Abstract

**Background:**

Perinatal social support and maternal education throughout the antenatal, childbirth, and postpartum phases contribute to the optimization of health and well-being of mothers and infants. Understanding deficits among support and educational resources can contribute to improved public health decision-making and maternal and child healthcare and wellbeing. This study aimed to explore health and social experiences and resources to characterize and unmet needs across the perinatal period.

**Methods:**

We conducted a qualitative study among 20 primiparous postpartum women from Punjab state, North India. Potential participants were identified from antenatal care registers maintained at healthcare sub-centers and were interviewed at their homes. Data were analyzed thematically, examining social support resources and needs across perinatal phases by social support domain: emotional, tangible, and informational.

**Results:**

Study participants received the most social and educational support antenatally. We identified considerably low knowledge surrounding childbirth and low maternal knowledge and social support around postpartum care and practices. Notable issues reported involved lack of consent, and satisfaction and comfort with childbirth care. Participant narratives highlighted the crucial role of both formal and informal support structures, with a heavy reliance on advice from community health workers (ASHAs), midwives, and doctors. Unmet needs were identified in areas such as post-cesarean section diet and care, institutional childbirth, and ASHA support, along with discrepancies between the advice given and evidence-based practices. While many participants' support needs were met by their networks, these gaps highlight critical areas for systems improvement.

**Conclusion:**

This study-identified gaps in maternal knowledge and support, particularly in the post-cesarean and institutional childbirth contexts, are underexplored in existing research. Findings suggest critical areas for improvement in maternal healthcare support, particularly regarding the alignment of advice with evidence-based practices. Programs should focus on educating women about the institutional childbirth process and providing better postpartum care education, especially regarding post-cesarean care and infant care. Strengthening the role of community health workers (ASHAs) with evidence-based training can improve support. Additionally, programs should enhance the integration of both formal (doctors, midwives) and informal support networks to create a more comprehensive care system.

**Trial registration:**

This research is part of the formative phase of a larger intervention trial. Trial was prospectively registered with Clinical trial registry of India. (CTRI/2020/12/029800 [Registered on: 15/12/2020].

## Background

Recent data estimates India’s maternal mortality ratio at 103 per 100,000 live births (2017–2019), representing an 81% percent decline from 556 per 100,000 live births in 1990 [1, 2]. Although maternal mortality rates have substantially improved over the last three decades, existing maternal health needs remain unmet. An analysis of India’s nationally-representative National Family Health Survey identified a significant increase in self-reported maternal complications between 2005–06 and 2015–16, from 43.6% to 53.7% [3]. While this increase may be due to both increases in occurrence and awareness, as a patient-reported outcome it emphasizes a need for broader attention to be paid to of maternal health care quality. High quality postnatal care and social support is associated with reduced maternal and neonatal mortality [4–6], and increased maternal engagement in behaviors promoting newborn (e.g., exclusive breastfeeding and child immunization) and maternal health (e.g., postnatal adoption of family planning) [7, 8].

Access to high quality care across the perinatal continuum is essential to prevent maternal and neonatal morbidity and mortality; however, important gaps exist and are patterned by socioeconomic status, raising equity concerns. A little over half (59.0%) of women achieve the Indian guidelines of four or more antenatal care (ANC) visits, with a notable difference between rural (55.0%) and urban populations (69.0%) [9]. Most births country-wide occur in health facilities (89.0%), though only 61.0% of mothers and 82.0% of newborns received a postnatal health check within 48 h of birth [9]. Women from the most affluent households (63%) are more likely to receive a postnatal check within two days compared to women from the lowest wealth quintile (58%) [10].

India’s broad variety of programs targeting maternal and neonatal health have successfully increased antenatal care and institutional childbirth. The National Rural Health Mission (NRHM; 2005–2012) created a robust community-based health workforce [9, 11], while Janani Suraksha Yojana (JSY) and Janani Shishu Suraksha Karyakram (JSSK) promoted institutional childbirth through cash incentives for public facility births, and provided free antenatal care, childbirth, postnatal care and neonatal care [9]. The SUMAN initiative (Surakshit Matritva Aashwasan) promotes access to respectful and high quality perinatal and newborn care [12]. These programs have increased institutional childbirth across all socioeconomic groups, and increased ANC usage across most states, reducing but not eliminating social inequities in perinatal care [13, 14]. India’s two million strong community health workforce launched within NRHM has played a key role in government program success through community-based service provision and health system linkage [9, 11, 15–17]. These Accredited Social Health Activists (ASHAs) have increased antenatal care, immunization coverage, and hospital childbirths, but were found less effective at postpartum and neonatal care, including counseling on nutrition and common neonatal and child illness prevention [13]. The high prevalence of gender inequitable norms including child marriage, restricted mobility, and low decision-making control has also influenced the success of programs targeting maternal and newborn health, with greater gender inequity limiting program impact [18].

Beyond care availability, high-quality perinatal care access is facilitated by health knowledge and social support [3, 19–21]. In certain Indian settings, knowledge of pregnancy-related danger signs is low [3, 20, 22], and misperceptions about infant illnesses is a major barrier to appropriate care-seeking [23, 24]. Social support across emotional, informational, and tangible domains is associated with health knowledge and care seeking, particularly when social support networks include educated females or health care providers [25–27]. Acknowledging the important role of health education and social support for optimizing perinatal health, we conducted a qualitative study to understand resources and gaps across the perinatal continuum of care in northern Indian women, providing a baseline understanding of maternal experiences and needs from the antenatal period through postpartum. Given the complexity and multifaceted nature of maternal health care in India, a qualitative study is the most appropriate design as it allows for an in-depth exploration of the nuanced experiences, perceptions, and social determinants impacting maternal health across different socioeconomic groups, thereby providing a comprehensive understanding of the resources and gaps in perinatal care from the perspectives of the women themselves.

## Methods

### Study overview and objective

Within a broader study to develop an mHealth intervention to improve maternal and neonatal health, intervention study [28], we conducted formative qualitative research among 20 postpartum North Indian women from Boothgarh block, Mohali district, Punjab, North India to understand their perinatal care perspectives and experiences, barriers to access, social support systems and informational resources, and needs for pregnancy and childbirth. The study objective was to conduct an in-depth exploration of maternal education, social support, and perinatal care within that context.

### Perinatal and newborn health in Punjab

Regional data from Punjab illustrate continued improvement in key perinatal and newborn indicators yet highlight areas of continued need. Perinatal care access reflects early antenatal care receipt (65.8%), a minimum of four antenatal care visits (59.3%), institutional births (94.3%), and maternal and child postnatal check-up by skilled health personnel within two days of childbirth (86.2% and 84.7%, respectively) [29]. Indicators of high-quality care, such as maternal consumption of iron and folic acid (40.5% for 180 days or more), anemia among pregnant women (51.7%), and postnatal checks beyond the first week of birth remain below recommendations [29]. Early (53.1%) and exclusive breastfeeding (55.5%)is low, and only 11.9% of children aged 6–23 are considered to have an adequate diet [29]. Among infants age 12–23 months, 76.2% are up to date on vaccination, yet care seeking for diarrhoea (67.5%) and acute respiratory infection (57.3%) is suboptimal [29]. Notable disparities persist across socioeconomic status and rurality [30].

### Study participants

Inclusion criteria were: primiparous and within 3 months postpartum. We prioritized primiparous women as they were experiencing pregnancy and childbirth for the first time and were likely to have greater education and social support needs. Potential participants were identified from antenatal care (ANC) registers maintained at Boothgarh’s 17 healthcare sub-centers using a convenience sampling approach to inform on priority health education and social support needs in this population. They were introduced to the researchers and study via telephone to ascertain interest in participating. Interested individuals were asked about their availability and scheduled for an in-person interview accordingly. The sample size for the study was determined by data saturation, monitored via a rapid analysis matrix [31]. No individuals invited to participate refused and no participants dropped out. All data were collected during one interview per participant and no follow-up with participants was conducted to review interview details or discuss findings.

### Data collection

In-depth interviews were conducted from October 2020 through December 2020, following India’s first major COVID-19 surge which peaked in September 2020. Data collection began as cases decreased and research restrictions allowed for in-person data collection with COVID safety protocols. Research staff (a qualitatively-trained PhD-level female interviewer PS and female note-taker MK) visited participants at their home, conducted the informed consent process including obtaining written confirmation, and conducted the interview face-to-face. The interviews were conducted in Hindi or Punjabi and lasted approximately 30–90 min. Interviews were audio-recorded upon participant permission and observation notes were taken; no participants refused permission for audio recording. The recorded interviews were simultaneously translated to English and transcribed for analysis.

The research team prioritized a quiet and private location; however, four interviews had a family member present for at least part of the interview. This included a mother-in-law briefly present at the beginning of the interview who left upon interviewer request, a sister who confirmed the interview purpose and intermittently re-entered during the interview, a sister-in-law who remained throughout the interview to provide comfort without interrupting the conversation, and a husband who occasionally joined the interview to assist with overcoming language barriers.

Interviews followed a semi-structured format. The interview guide was informed by investigators’ prior research and small iterations were made during the study to improve understandability and flow. The interviewer (PS) was well versed with the interview guide to keep the environment of the interview as informal as possible. The note-taker (MK) recorded important observations such as attitude and behavior of the participant, important quotes, timing of the interview, location details, etc. The interview guide focused on women’s experiences during their recent pregnancy, at childbirth, and postpartum. Interviewees were asked to share their views on perinatal care, pregnancy and postnatal care in the community, barriers to accessing postnatal care, existing social support systems after childbirth, and information needs in the postnatal period. We also sought information to inform a mobile health education intervention to improve maternal health outcomes.

### Data analysis

We employed thematic analysis using both deductive and inductive approaches. Transcripts were coded using Dedoose qualitative analysis software (Los Angeles, CA, USA) following a codebook developed from the in-depth interview guide and iteratively supplemented by unexpected findings in the data as identified. The analysis remained flexible, allowing for the emergence of unexpected themes through inductive coding. This approach enabled us to incorporate both pre-determined categories based on the interview guide and novel insights that surfaced during the analysis, providing a more nuanced understanding of the data.

After jointly coding 4 transcripts together to standardize coding, our team of 5 Indian (PS, MK, AA, AK, DB) and 3 American (ND, RP, AE) researchers coded the transcripts independently, addressing queries and discrepancies through regular group discussion. The Indian researchers were familiar with the local context, which provided valuable cultural insights, while the involvement of American researchers offered an external perspective. The joint coding process including the team’s ongoing discussions helped ensure a balanced and objective analysis. This approach aimed to enhance the rigor and reliability of the findings while being mindful of the researchers’ backgrounds and potential influences on the data collection and analysis process.

The study focused on social support and health education across the perinatal period, employing Berkman and Glass’ social support framework which provides a conceptual framework for considering how social-structural conditions shape social networks which influence psychosocial mechanisms and impact health through behavioral, psychological and physiologic pathways [32]. Our analysis employed three of the four key social support domains identified by Berkman and Glass to classify the types of ways in which health status may be influenced by key individuals in a woman’s life: emotional, tangible, and informational support [32]. Emotional support refers to empathetic interactions, offering reassurance, comfort, or acceptance. Tangible support refers to contributing resources, finances, or assistance. Informational support refers to knowledge, facts, and advice or feedback on actions. Data were analyzed within broad perinatal timeframes (i.e., antenatal, childbirth, and postnatal).

## Results

### Participant characteristics

Participants ranged in age from 20–31 years (Table 1). Most (*n* = 16) had completed higher secondary school or above. Three had pursued diplomas and one participant had no schooling. All participants except one lived in multigenerational households including in-laws, brothers and sisters-in-law, and their children. All participants were married, except for one who was recently widowed. No participant lived in their natal village. Husbands’ occupation varied (e.g., government job, car driver, plumber, auto driver, mechanic, farmer, etc.); two women worked before childbirth (lab technician, boutique owner) but all were non-working at the time of interview.

**Table 1:** Sociodemographic characteristics of study participants (*n* = 20)

Characteristic	N (%)
Age^a^	25.5 (22.5–28)
Education	
No schooling	1 (5%)
Primary School	3 (15%)
Secondary School	11 (55%)
Some Secondary School	2 (10%)
Other	3 (15%)
Able to read	
Cannot read	1 (5%)
Can partially read	3 (15%)
Able to read	16 (80%)
Able to write	
Cannot write	0 (0%)
Can partially write	3 (15%)
Able to write	17 (85%)
Relationship status	
Married	19(95%)
Widow	1 (5%)
No. of children^a^ (of the participant)	1 (1–1)
No. of children in the household^a^	2 (1–3)
No. of adults in the household^a^	6 (4.75–7)
Do the parents live in the same village?	
No	20 (100%)
Monthly income husband,^a^ Indian Rupees	
0–10,000	2 (10%)
10,001–20,000	9 (45%)
20,001–30,000	0 (0%)
30,001-above	2 (10%)
Did not share	7 (35%)
Monthly income woman,^a^ Indian Rupees	0 (0–0)
Not working	20 (100%)
Ration Card category	
Orange (Below the poverty line)	1 (5%)
Yellow (Above poverty line)	2 (10%)
Do not know	17 (85%)
Household phone ownership	
Yes – smart phone	20 (100%)
Personal phone ownership	
Yes – smart phone	13(65%)
Yes – feature phone	7 (35%)
Access to household phone	
Daily	12 (60%)
Weekly	0 (0%)
Less than weekly	8 (40%)

^a^Median (IQR)

### Social support resources and needs by perinatal phase

**Fig. 1:**
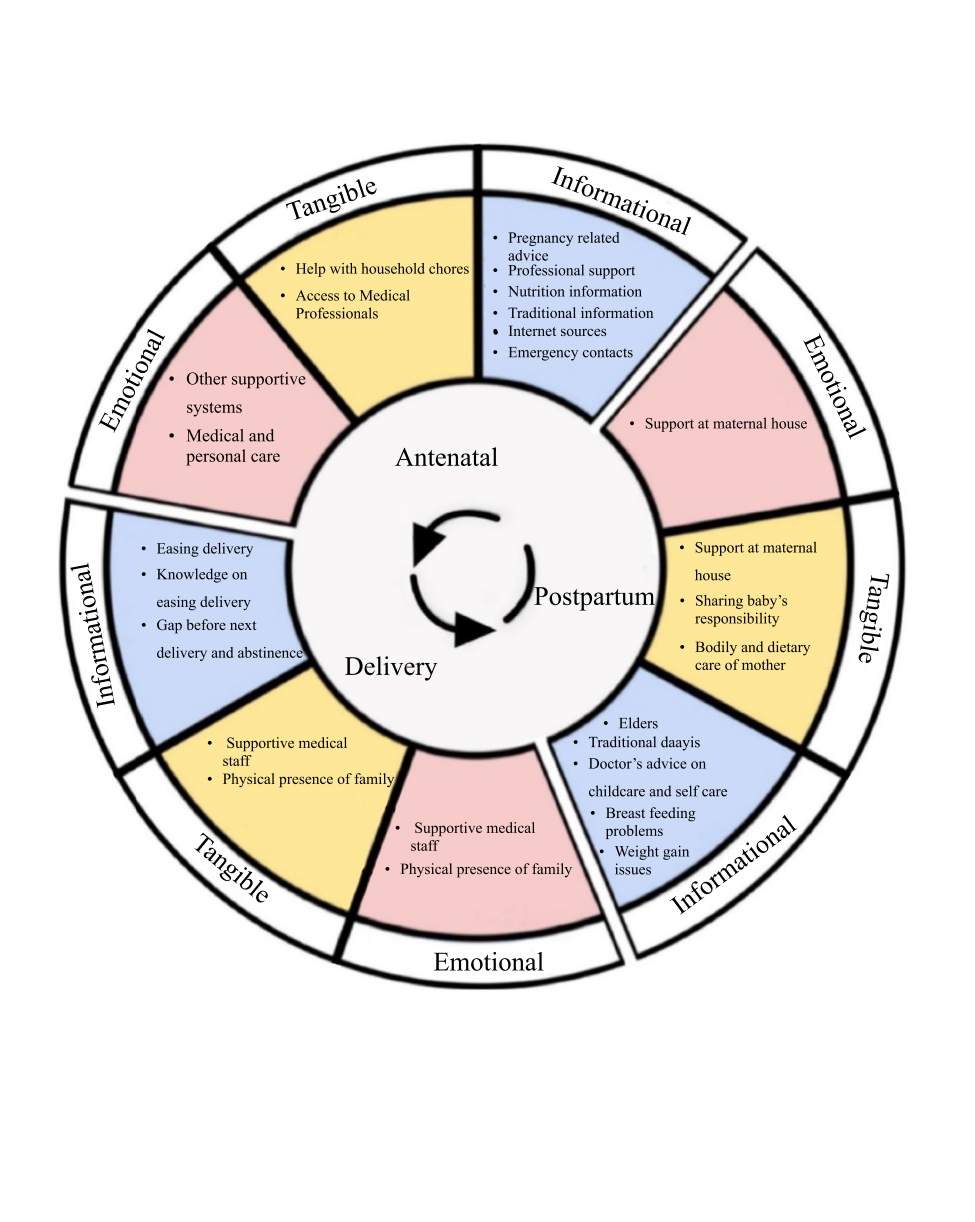
Summary of themes by social support domain and perinatal continuum of care stage

Participant narratives were analyzed for social support resources and needs during each phase of the perinatal period and themes were organized by major social support domains; emotional, tangible, and informational [31]. Figure 1 presents the theme summary by social support domain and perinatal continuum of care stage.

#### Antenatal

##### Emotional support

Emotional support from neighbors, aunts, relatives, sisters-in-laws, friends, and especially from older women who already had a baby largely focused on stress relief.

*“All [family members] used to say don’t take tension (be afraid). In the first pregnancy, women get scared at the time of delivery, so they told me not to take tension, keep calm. Otherwise, there will be a problem with normal delivery.”- 25 years old, ‘other’ educational attainment, 1 child, C-Section*

##### Tangible support

Participants reported various tangible support antenatally, including help with household chores, and facilitation of medical and personal care. While most participants typically bore the primary responsibility for household work such as washing clothes, cleaning, and cooking, some respite was provided during pregnancy by mothers-in-laws and sometimes husbands. This was motivated by perceived miscarriage risks. Newly pregnant women were generally limited to lighter work such as folding clothes, cutting vegetables, and dusting. They resumed a wider task range during the second trimester. Interestingly, in the third trimester, activities such as mopping floors were considered beneficial. This is rooted in traditional beliefs that such activity can widen the pelvis, increasing the likelihood of successful vaginal childbirth.

*“My husband was a big support [throughout my pregnancy], he did all household chores like washing clothes, brooming and I used to cook only.” – 24 years old, secondary school or higher, 1 child, living with husband and father-in-law, Vaginal childbirth*

Family members played a crucial role in supporting pregnant women especially in accessing medical care, diet, and rest. Participants emphasized the vital importance of their family members in facilitating their access to the medical care needed for a healthy pregnancy. All participants were from rural areas, where transportation was limited. Moreover, social restrictions often restrict women from traveling to the hospital alone. To address these challenges, husbands drove women to their antenatal visits. Husbands focused on women’s diet during pregnancy, regularly bringing fruits and ensuring they ate frequently. One woman mentioned her husband’s guidance to avoid fried food. Another described her husband’s attention to her physical health throughout pregnancy. Family members also contributed to the pregnant women’s comfort and well-being, for example, husbands giving her massages to alleviate pain and mother-in-laws offering home remedies for pregnancy-related discomforts, such as stomach aches, bodily pains, and morning sickness.

*“I don’t pay much heed about eating so [my husband] usually fed me. Sometimes he bought apple and sometimes other fruits. He used to cut and fed them to me. I wasn’t well at that time, I would keep lying on bed. He would wake me and feed me. I didn’t take as much care of myself as he took.” – 22 years old, secondary school of higher, 1 child, Vaginal childbirth*

Participants reported having access to highly supportive and readily accessible primary medical care in their villages including ASHAs, Auxiliary Nursing Midwives (ANM), Community Health Officers (CHOs), and physicians at a dispensary or private clinic. Most women had registered at the nearest public health facility during the first trimester and were given a mother and child protection card to track pregnancy details. The card explains the required antenatal checkups, biomarkers to be monitored, pregnancy danger signs, etc. Throughout their pregnancies, the women regularly accessed antenatal care. They found medical professionals to be approachable, with some even providing their phone numbers for emergencies. A widowed participant emphasized the importance of mobile phones for facilitating home-based pregnancy care, especially given her reduced support for physical access. However, women without provider telephone access had not perceived this as a need; they had the support they needed for a healthy pregnancy regardless. Overall, participants were satisfied with the medical support provided by the sub-centers, private professionals, and tertiary health care system.

*“Yes, I had bleeding in the 5*^*th*^* month, it wasn’t much, but I had bleeding. I was at my parental home, then I consulted doctor on the phone, and they gave me medicine for 5 days. They said it is normal, it happens, and there is nothing to fear. Whenever it happens, advise should be taken [from a doctor].” – 28 years, tenth standard, lives in a joint family*

*“I think it is essential to have a doctor’s contact number [during pregnancy]. Especially when your husband is no more because with a spouse it is much easier for a woman to manage during pregnancy. And one thinks that if we have phone in our life then we should make some use of it. We have to use our mind and have to depend on ourselves. When God makes you go through such situations in life then you have to do and learn many things. Life teaches you everything.” – 30 years, some secondary school education, 1 child, C-section*

##### Informational support

Participants actively sought from family and others. They sought day-to-day advice from mothers-in-law, sisters with children, and other elders. They regularly communicated with community health workers and providers for medical queries, and regularly used the internet and YouTube videos for education. Informational needs voiced included how to have a healthy pregnancy, an appropriate diet, and how to solve problems. Mothers and mothers-in-law heavily influenced the food that women ate and avoided during pregnancy.

*“Mostly, I would ask my sister and sisters-in-law. Since my husband has two elder sisters, I would ring them up and ask about my problems. Slowly-slowly I would collect all the information from everywhere and got to know what is right and what is wrong then I would apply accordingly. For example, if the thing matched between three people, I would do that.” – 25 years old, ‘other’ educational attainment, 1 child, Vaginal childbirth*

*“During pregnancy my mother had told me not to eat anything that is hot in nature, especially in the beginning. In the initial months she used to give me healthy things like milk and curd, fruits like pomegranate, apples, etc. My mother used to ask me to eat these things more.”- 26 years old, primary school, 1 child, living in a joint family,* Vaginal *childbirth*

Elders played a significant role in providing information to pregnant women in multi-generational households. This advice was taken very seriously and included conventional practices, e.g., ways to channel positive energy, specific foods for a healthy pregnancy, postures, and activities to increase the likelihood of a vaginal childbirth. One recommendation was mopping the floor to facilitate vaginal childbirth. Elders categorized foods into ‘hot’ and ‘cold’. Women were guided not to eat papaya, pineapple, eggplant, dry fruits, and other ‘hot’ foods in the first three months of pregnancy due to miscarriage fears.

*“Typically, I would listen to religious hymns only and would listen to Bhajans (religious songs). Also, the elders would say the more you listen to religious things, the more it will give positive effects on the baby.”- 25 years old, ‘other’ educational attainment, 1 child, Vaginal childbirth*

Community-based ASHA workers were the primary health contact for pregnant women. They provided information about available medical care and shared pregnancy-related information, including immunization, healthy diet, and supplements. Women reached out to their ASHA workers with questions, and appreciated their support during the COVID-19 lockdown, when clinics were closed and doctors were unavailable.

*“[I was in touch with my] ASHA worker only because due to COVID, clinics were not open. Some tests were done and some were not able to be done. This happened all the time due to COVID. And sometimes I visited Mohali Hospital. All my tests were done there…. [My ASHA] explained very nicely and told me to contact her in case of any problem. She lives nearby [my house], and is very supportive. She explained medicines to me…. I used to get sick at that time. She would counsel on eating healthy, and staying fit.” – 22 years old, secondary school or higher education, 1 child, Vaginal* childbirth

Some women maintained contact with doctors via phone. Those registered at private clinics received reminder calls for scheduled check-ups. Women also visited doctors for medical care, which included routine checkups, scans, and discussions about other health issues.

*“I used to phone call my brother, and he used to ask the doctor about the problems I had. Sometimes I had pain in the lower abdomen; they suggested avoiding lifting weight.”- 28, tenth standard, lives in a joint family, C-section*

*“[During the third trimester], Doctors used to call and we used to go every week. The baby keeps moving and we get to know that the baby is fine. They also used to come to check the heartbeat to see if it is fine or not.”- 26 years old, primary school, 1 child, living in a joint family, Vaginal childbirth*

Women shared various maternal and fetal health concerns for which they sought medical advice. ASHA workers educated women on healthy everyday practices, such as diet. Doctors offered constant support in case of complicated pregnancies, such as twin pregnancy. Other queries focused on medication to be taken during pregnancy.

*“The doctor [at the hospital] was very nice. She used to talk to me very politely. Whatever she used to ask, I used to tell her. Like I used to feel pain in the lower region, I used to tell her. She used to say this much will happen. It should not be much, but little bit will happen. Just that. Overall, doctors were good there.”- 26 years old, primary school, 1 child, living in a joint family, Vaginal childbirth*

Most participants owned smartphones, and commonly used the internet, particularly Youtube videos, for pregnancy-related queries. Common searches included videos on nutrition, sleeping postures, and determining baby’s sex. Educational videos were the primary source of educational content. Women’s narratives demonstrated how crucial the internet was in providing antenatal support, which they accessed independently of professional guidance.

*“I used to find out if it’s a girl or boy by watching the scans…(laughs). They used to show signs in the videos, if this sign is girl. That sign, it is boy….(laughs)” – 31 years old, secondary school or higher, 1 children, Vaginal Childbirth*

*“Yes, I have used the phone to see how to sleep while pregnant… like when I will sleep, there should not be any problem to the baby. In fact, I have seen how to tell whether there is any problem or not on YouTube. I have seen how to sleep, how to sit, how to eat, and what to eat. Eat vegetables and fruits, and drink juice.” – 20 years old, ‘other’ educational attainment, 1 child, Vaginal Childbirth*

#### Childbirth

##### Emotional and tangible support

Participant narratives intertwined emotional and tangible support around childbirth. They discussed individuals providing both emotional and tangible support, and the tangible support provided to assist women’s birthing experiences.

When discussing their birthing experiences, participants mentioned the support they received from their husbands, brothers, mothers, and mothers-in-law. These individuals provided emotional support and practical assistance such as coordinating with hospital staff for prescribed essentials and medications. Family members, especially mothers, kept the women calm during labor. Male relatives, though not allowed in labor rooms, assisted by purchasing prescribed medicines and running errands. All participants expressed gratitude for this support. One woman expressed how her husband’s communication with the nurse comforted her:

*“Yes, [my husband] said nothing will happen. He kept on talking to the nurse that nothing should happen to both of us (baby and the participant). Both should be fine.”- 22, 9*^*th*^* standard, lives in a joint family, child 1, C-Section*

Women reported feeling comforted by the support of medical staff during childbirth, highlighting the sense of security provided by trained medical professionals in hospitals who can quickly respond to emergencies and offer continuous care, contrasting with the perceived lack of professional support at home:

“*If anything happens, then the nurse is there to look after you at the hospital, but at home there is no one to look after you.”−18, Primary School, lives with husband, child 1, vaginal childbirth*

Narratives revealed the key importance of tangible social support during labor and birth, particularly where participants’ lack of knowledge around emergency medical procedures challenged informed consent. For example, two participants reported discomfort with having to rapidly decide about cesarean surgery with no knowledge.

Sometimes, family members were not permitted with the women during childbirth but women reported feeling emotionally supported by nurses when making hard decisions. In one instance, a nurse comforted the participant who was undergoing cesarean surgery, further highlighting the compassionate role of medical professionals during these challenging moments.

*“The doctors and nurses were nice. The doctor who operated was nice. They all talked with me during the operation, kept me calm all the time.” - 25 years old, ‘other’ educational attainment, 1 child, C-Section*

However, not all women reported positive experiences with hospital staff. One participant described a challenging interaction during her labor, stating: *“They were telling me to lay down so that I can get contractions, but I was restless. They kept scolding me because I wasn’t sitting. Then after some time, I delivered. It was very difficult. They kept pushing the baby. After delivery they gave me stitches.”−27 years old, secondary schooling, 1 child, vaginal childbirth.*

##### Informational support

All participants were first-time mothers who felt anxious about giving birth and sought information to prepare themselves. However, they generally lacked knowledge about the birthing process. Their mothers usually comforted them without sharing critical details to avoid causing fear. Women also sought knowledge from their mothers, friends, and sisters on practices to ease childbirth, such as eating certain foods or doing certain activities. Mothers shared tips on pushing techniques, and recommended foods that would help alleviate childbirth pain. While not evidence based, this support helped reduce women’s anxieties around labor and birth.

*“Everybody used to tell me to eat well, and also to keep working so that I have a normal delivery. Sometimes the private doctors suggest bed rest, as they suggested to me when I had pain. So, I used to rest. But everyone used to suggest working to have a normal delivery. I used to go for a walk in the evenings as much as possible, because this will help in normal delivery.”- 26 years old, primary school, 1 child, living in a joint family, vaginal childbirth*

“*My mother-in-law would tell me that at the time of delivery, I should push with all the force and not take too many deep breaths… like this [enacts the action] … this can move the baby backwards.”−41 years old, vaginal childbirth*

Seven participants underwent cesarean sections, while 13 had vaginal childbirths. The women’s narratives revealed a significant lack of education about cesarean section. In many cases, women only learned of the possibility of a cesarean section on the day they were admitted to the hospital which made emergency decision-making difficult.

*“When I was in the 9*^*th*^* month, I was scared about what will happen. They would tell me that in normal delivery, there is a lot of pain and the person can’t handle it. That there is a lot of difficulty during that time. In the last part, I had difficulties… that’s it.” – 31 years old, secondary school education, 1 child, C-Section*

#### Postpartum

##### Emotional support

Women’s accounts of postpartum emotional support centered around recovery from childbirth. Some participants moved to their natal homes during the third trimester of pregnancy, whereas others did so after giving birth. In their natal homes, women benefitted from support from their parents, and felt comfortable in familiar surroundings. They felt relieved of all household responsibilities and could physically rest while their mothers cared for their babies.

*“At my mother’s place, there was no such work to do. Your mother does the work, and so does your sister-in-law. For one month, one aunt came to bathe the baby… we had hired her. After that I started doing it myself... for the baby and for myself too. Clothes and all, mother used to wash.” – 30 years old, some secondary education, 1 child, Vaginal childbirth*

##### Tangible support

During the postpartum period, new mothers faced dual challenges: adapting to their own bodily changes and the responsibilities of newborn care. Families aided in caring for both women and their babies. Women mentioned movement restrictions for the initial few weeks due to stitches; nearly all participants had some intervention (seven underwent cesarean section and 13 underwent episiotomy). All were first time mothers and therefore were nervous with baby care. In most cases, responsibility for the child was taken by their mother-in-law or mother. This included bathing, cutting nails, giving massages, etc. Watching their mothers or mothers-in-law care for the baby helped women learn and gain confidence. Women staying at their natal home appreciated this caring environment. Respondents also appreciated involvement of husbands and other family members in occasional childcare care duties:

*“This is my first child, so I don’t know much about caring. My mother-in-law does everything. I usually do what she suggests. I keep asking her. She would make me sit and teach me how to give a massage to baby.” – 22 years old, secondary school or higher education, 1 child, Vaginal childbirth*

*“During the night [my husband] keeps the baby with him when I get tired. In the evening, he also keeps holding her, and goes around the house with her. He also plays with her during the night when I get tired. I cannot sit for a long time.”- 26 years old, primary school, 1 child, living in a joint family, vaginal childbirth*

Culturally, postpartum women are advised substantial rest and nutritious diet to support recovery. Women who had vaginal childbirth were encouraged to eat panjiri, a high-calorie snack made of ghee, nuts, dried fruits, sugar, and herbal gums, to improve lactation and warm the body. Both mothers-in-law and mothers prepared panjiri for the new mothers. However, doctors advised women who had cesarean section to avoid panjiri, as high calorie foods are believed to slow wound healing.

*“My mother gave me panjiri, which is rich in desi ghee and dry fruits. This is so that the baby’s health improves. It will benefit the baby.” – 24 years old, secondary school or higher education, 1 child, vaginal childbirth*

Women mentioned physical discomforts after childbirth, including pain from stitches, weakness, and leg pain. Mothers and mothers-in-law helped the women sit, assisted with breastfeeding, washing, massages, and other needs to increase comfort. Participants felt this support contributed to their faster recovery. In one case, woman who lived alone with her husband had a sister stay to provide postpartum care.

*“I was good, my mother would help me in sitting and standing. My younger brother helped my mother in taking care of me.”- 28, tenth standard, lives in a joint family, C-Section*

*“I take rest, or [my sister-in-law] massages my legs and arm, as massage cannot be done on abdomen because of stiches.” – 25 years old, ‘other’ educational attainment, 1 child, C-Section*

##### Informational support

Informational support needs paralleled the infant and self-care described within emotional support. In addition, as the women recovered and resumed sexual intercourse, they considered contraceptives. New mothers sought childcare advice from various sources, including their mothers, sisters, neighbors, ASHA workers, and other family members. Primary concerns included baby massage, foods to eat to promote baby health, and superstitious practices to ward off evil. Some mothers firmly believed the information shared with them and religiously followed the advice. They were certain that those with previous childcare experiences would provide valuable guidance. Additionally, women sought informational support from ASHA workers about child vaccination.

*“Actually, I feel happy if somebody guides me, tells me what is right and wrong, and advises me as I am new to this. I feel happy if somebody informs and I don’t feel that somebody is interrupting me. I feel happy that before a mishappening, I have caught the problem and have taken preventive measures. Like somebody told me if I ate too hot food, it would affect the health of the baby; likewise, if I ate sweet food then the baby will have saliva leaking, and if I consumed spicy food then the baby might suffer from stomach pain. I want somebody to tell me what is good or bad for my baby and me.” – 25 years old, ‘other’ educational attainment, 1 child, Vaginal* childbirth

Other mothers were often a valuable resource for informational support for self-care. For instance, one mother experienced breastfeeding challenges, and a new mother in the hospital advised her on formula feeding as an alternative. Similarly, women experiencing pain from perineal and abdominal stitches, sought guidance from doctors and fellow mothers.

*“There was another [mother] who had delivered her baby there. She was also having pain in the breast, and she told me that she also feed the baby with that powder...she said that she bought it from here. Then we fed it one day only, then never gave that again. I am giving [breastmilk] only, despite the pain.”- 27, 10*^*th*^* Standard, lives in a joint family, 1 child, Vaginal* childbirth

**Postpartum contraception**

Most respondents targeted a specific gap between pregnancies. Some had consulted doctors or family members for family planning education; one woman also mentioned receiving contraceptive counseling at the hospital, which covered intrauterine devices, oral contraceptive pills, and condoms. A few women, though unaware of contraceptive methods, expressed a desire to delay pregnancy.

#### Effects of the coronavirus pandemic on seeking care

The coronavirus pandemic impacted respondents perinatal experiences, imposing various limitations. Movement restrictions prevented women from visiting their maternal homes, potentially affecting their mental health. Lack of mobility and transportation hindered access to everyday necessities. Additionally, participants also mentioned missing some check-ups due to closures in response to the pandemic.

*“I went every month for check-up…. I was scared because it was corona time and nothing should go here and there. After that, I would go for regular check-ups, like blood tests and BP check-ups.” – 20 years old, ‘other’ educational attainment, 1 child, Vaginal childbirth*

*“Yes. It happened only once, and then it didn’t happen because of corona. Everything was closed because of that. Check-ups were not done.” – 20 years old, ‘other’ educational attainment, 1 child, Vaginal childbirth*

## Discussion

### Interpretation of major findings

Participant narratives describe the important role of formal and informal support structures in addressing maternal health needs. Participants had strong informal support systems consisting of husbands, family members, friends, and neighbors. Most participants reported adequate healthcare access. Women also sought formal community health support in their community including ASHA workers, midwives, and doctors. While many of the participants’ support needs were met by their support networks, our study identified certain areas where support needs remained unmet, in addition to discrepancies between the content provided and evidence-based care. Participants’ narratives show that for the most part, their emotional, tangible, and informational support needs during the antenatal period were met but larger gaps existed during childbirth and postpartum. These findings are consistent with other research from diverse Indian settings which have confirmed greater needs for informational and emotional support at the time of childbirth [33, 34].

A major concern of the informational support reported in this study was focused on the family member source, both marital and natal, and the fact that multiple key themes were not evidence-based. This finding suggests ongoing tension between sociocultural norms and medical evidence. While sociocultural traditions are valued have been found to provide comfort perinatally [35], they may also negatively influence maternal and infant health [36–38]. Literature from the Indian setting on this topic describes many key sources of informal informational support as inadequately educated on antenatal maternal care and danger signs, including husbands [39–41], who are likely to be key perinatal care decision-makers [42, 43]. Additionally, information sought by our study participants about traditional practices to help ease pregnancy-related problems is in line with other Indian literature on illness perceptions and cultural beliefs which prevented care-seeking. This includes traditional beliefs on nutrition and food consumption, herbal medicines, the role of family and husbands, and antenatal rituals [23, 30]. Similar beliefs were prevalent in our study, signifying the importance of healthcare providers and community health resources in overcoming prevalent yet incorrect practices. This is especially important considering they are highly dependent on informational support from medical professionals for medical needs. ASHA workers and doctors played a significant role in supplying pregnant women with informational support regarding sickness and pregnancy-related complications. These results were expected, as ASHA workers and providers primarily assist women antenatally [15–17].

Emotional support at childbirth was provided by family members to instill confidence, and medical staff to help women give birth easily. Women’s fear of labor pains and information-seeking from both informal and formal networks on ways to ease pain was similar to previous Indian literature where women obtained most childbirth information from family and friends [44]. While government-funded programs have successfully increased facility birth to 80% nationally [45], these formal networks have been less successful in educating women about childbirth, as noted by our study participants’ lack of knowledge. This is consistent with other research from India which reports only 27.7% of participants had knowledge of labor and 21.2% of sufficient knowledge of childbirth [20], and led to our participants having to make emergency childbirth decisions without prior information, presenting challenges for understanding and informed consent. The lack of childbirth and postpartum knowledge may contribute to adverse maternal health, as 66% of maternal mortality occurs around this time [2]. This lack of knowledge with regards to childbirth must be targeted to provide pregnant women with the information they need to make informed decisions during childbirth.

During the postpartum period, women had significant emotional and tangible support needs for caring for themselves and their infants, and they relied heavily on their family members. Additionally, lack of proper support for neonatal and postpartum health issues by ASHA workers in comparison to antenatal care indicates more postpartum support from formal networks is needed that can provide clear evidence-based information [13]. Women were confused by contradicting postpartum dietary advice between doctors and their elders. Previous studies have also identified misconceptions regarding the c-section diet and wound healing among providers, families, and women. Many misconceptions are not aligned with evidence-based guidance on diet, which consists of postpartum women intaking an extra 500 kilocalories, encouraging food intake within two hours after cesarean section, and a complete calorie dense diet within eight hours of surgery, thereby improving recovery [46]. In regard to postpartum contraception, women need informational support. Husbands’ perspectives, missing from our interviews, play a crucial part in contraceptive decision-making.

### Recommendations for formal and informal support networks

The knowledge gaps found among our study participants suggests disparities in accessibility to maternal health knowledge in rural areas. This aligns with previous studies, as significant variations have been found in the level of continuum of care across states and socioeconomic groups of India, indicating limited care accessibility among less economically advanced regions [14]. Additionally, government-funded programs such as the JSY and ASHA workers focused on institutional childbirth and antenatal care [11, 12, 16] may not have been as effective in increasing maternal knowledge, consistent with their healthcare access goals [9, 11, 13]. Given these findings, government-funded programs should further address maternal knowledge gains, particularly at the community level where impact on sociocultural norms and broader network knowledge may have greater influence [38].

Although formal care networks have substantially improved perinatal care, our results indicate major gaps in informational support. Across the perinatal continuum, women relied more heavily on informal networks consisting of husbands, family, and neighbors which is consistent with nationally-representative studies which found that 62.5% of women make healthcare decisions in conjunction with their husbands, and 15.3% of women did not receive antenatal care due to husbands not feeling it was necessary [10]. Previous studies showed better spousal relationship quality was linked to antenatal care and facility birth, and similarly where women in joint families had better relationships with their in-laws [47]. National Indian data also identified gaps in antenatal counseling of partners, with only 63.9% of men who attended ANC visits counseled on what to do if their wife had pregnancy complications. Programs related to maternal care should account for the familial networks that women have, as this heavily influences their decision-making with regards to health concerns. Interventions for husbands and other familial support can be designed to better support the needs of pregnant women.

#### Implications for practice and future studies

The government of India’s diverse programs to improve perinatal and neonatal health include Pradhan Mantri Surakshit Matritva Abhiyan (PMSMA), which consists of free antenatal care once per month, and the Janani Shishu Suraksha Karyakaram (JSSK), which consists of free maternal health services. Gaps in accessibility and knowledge preventing the overall quality and continuum of maternal health care in India can be addressed through improving the educational offerings of social programs with evidence-based guidance [9]. Pregnant and postpartum women may also benefit from educational programs designed for their informal networks, including family and neighbors, to better deal with pregnancy and postpartum issues. Furthermore, women would benefit from communicating with one another on pregnancy-related issues, as important information can be exchanged through social networks, and subsequent research and programming should also take advantage of our participants’ use of online education sources, particularly given other literature on the acceptability and potential impact of digital resources [48–52]. Programs could build in-person or mHealth groups relying on digital devices for support. A focus on improved knowledge among both informal and formal support networks is essential for assisting pregnant women with the perinatal continuum, given the importance of social network guidance.

### Strengths and limitations

This qualitative study provided nuanced information on health education and social support needs among a select group of rural Indian women. While the results may not be generalizable due to our convenience sampling approach from among women already engaged with formal healthcare services, these findings describe a range of potential concerns important for maternal health research and practice. Our study was strengthened using a social support framework which allowed for a robust theoretical classification of support needs. Our study’s attention to both informal and formal sources of support provides a comprehensive analysis of the diverse support resources that women depend on. Finally, to improve interpretation of qualitative findings, the analysis and interpretation of these data employed a team approach.

Limitations to our study include the aforementioned lack of generalizability of our qualitative sample concentrated on first-time mothers within a smaller geography with health-care access. It is worth noting that the focal community is generally well-connected to the formal healthcare system, as the government provides these services free of charge and thus a significant proportion of the population is likely to have engaged with the formal healthcare system. The presence of family members in four interviews may have affected what the participant shared during the interview but was unavoidable due to sociocultural norms and logistics. Our findings are limited by incorporating only one perspective on this topic; other studies incorporating diverse perspectives of other stakeholders such as those of spouses, mothers-in-law, and healthcare providers would help to strengthen our understanding.

## Conclusion

The results from this study suggest that ongoing efforts to reduce maternal and neonatal morbidity and mortality in India must strive to overcome important gaps in informational support needs among pregnant women and their households, particularly around childbirth and postpartum. Broadly improving perinatal knowledge is likely to require multiple strategies across community to facility levels, and may encompass diverse modalities to achieve scale. In combination with India’s broad efforts to improve perinatal care access, addressing key social support needs is likely to improve maternal and neonatal health and wellbeing.

## Data Availability

The datasets used and/or analysed during the current study are available from the corresponding author on reasonable request.

## Abbreviations

ANC: Antenatal care
ASHAs: Accredited Social Health Activist workers
JSY: Janani Suraksha Yojana
JSSK: Janani Shishu Suraksha Karyakram
NRHM: National Rural Health Mission
SUMAN: The Surakshit Matritva Aashwasan

## References

[1] Office of the Registrar General & Census Commissioner, India. India - Sample Registration System (SRS)-special bulletin on maternal mortality in India 2017–19. https://censusindia.gov.in/nada/index.php/catalog/40525. Accessed 6 Sep 2022.

[2] WHO, UNICEF, UNFPA, World Bank Group, United Nations Population Division. Trends in maternal mortality: 1990 to 2015. Geneva: WHO, UNICEF, UNFPA, World Bank Group, and United Nations Population Division; 2015. http://apps.who.int/iris/bitstream/10665/194254/1/9789241565141_eng.pdf.

[3] Kumar G, Choudhary TS, Srivastava A, et al. Utilisation, equity and determinants of full antenatal care in India: analysis from the National Family Health Survey 4. BMC Pregnancy Childbirth. 2019;19:327. 31488080 10.1186/s12884-019-2473-6 PMC6727513

[4] Fadel SA, Ram U, Morris SK, et al. Facility delivery, postnatal care and neonatal deaths in India: nationally-representative case-control studies. PLoS One. 2015;10:e0140448. 26479476 10.1371/journal.pone.0140448 PMC4610669

[5] Say L, Chou D, Gemmill A, et al. Global causes of maternal death: a WHO systematic analysis. Lancet Glob Health. 2014;2:e323–33. 25103301 10.1016/S2214-109X(14)70227-X

[6] World Health Organization. WHO recommendations on postnatal care of the mother and newborn. Geneva: World Health Organization; 2013 https://www.who.int/publications-detail-redirect/9789241506649. 24624481

[7] ACOG Committee Opinion No. 736: Optimizing Postpartum Care. Obstet Gynecol. 2018;131(5):e140-50. 10.1097/AOG.0000000000002633. 10.1097/AOG.0000000000002633 29683911

[8] Dixit P, Dwivedi LK, Gupta A. Role of maternal and child health care services on postpartum contraceptive adoption in India. SAGE Open. 2017;7:2158244017733515.

[9] Bhatia M, Dwivedi LK, Banerjee K, Bansal A, Ranjan M, Dixit P. Pro-poor policies and improvements in maternal health outcomes in India. BMC Pregnancy Childbirth. 2021;21:389. 34011316 10.1186/s12884-021-03839-w PMC8135986

[10] Ministry of Health & Family Welfare, Government of India. National Family Health Survey (BFHS-5) 2019–21 compendium of fact sheets: key indicators, India and 14 states. New Delhi: Ministry of Health & Family Welfare, Government of India; 2022 https://mohfw.gov.in/sites/default/files/NFHS-5_Phase-II_0.pdf. Accessed 1 June 2023.

[11] Vellakkal S, Gupta A, Khan Z, et al. Has India’s national rural health mission reduced inequities in maternal health services? A pre-post repeated cross-sectional study. Health Policy Plan. 2017;32:79–90. 27515405 10.1093/heapol/czw100 PMC5886191

[12] Ministry of Health and Family Welfare, Government of India. SUMAN | Surakshit Matritva Aashwashan. https://suman.nhp.gov.in/. Accessed 23 Jan 2023.

[13] Fathima FN, Raju M, Varadharajan KS, Krishnamurthy A, Ananthkumar SR, Mony PK. Assessment of ’accredited social health activists’-a national community health volunteer scheme in Karnataka State. India J Health Popul Nutr. 2015;33:137–45. 25995730 PMC4438657

[14] James K, Mishra U, Pallikadavath S. Sequential impact of components of maternal and child health care services on the continuum of care in India | Journal of Biosocial Science | Cambridge Core. J Biosoc Sci. 2021;54:1–23.

[15] Ministry of Health and Family Welfare. Accredited Social Health Activist (ASHA) guidelines. https://nhm.gov.in/index1.php?lang=1&level=1&sublinkid=150&lid=226.

[16] Mishra A. ‘Trust and teamwork matter’: community health workers’ experiences in integrated service delivery in India. Glob Public Health. 2014;9:960. 25025872 10.1080/17441692.2014.934877 PMC4166967

[17] National Rural Health Mission. ASHA-which way forward? Evaluation of the ASHA Programme. New Delhi: National Health Systems Resource Centre (NHSRC); 2011. http://www.nipccd-earchive.wcd.nic.in/sites/default/files/PDF/Evaluation_of_ASHA_Program_2010-11_Report.pdf.

[18] McDougal L, Atmavilas Y, Hay K, Silverman JG, Tarigopula UK, Raj A. Making the continuum of care work for mothers and infants: Does gender equity matter? Findings from a quasi-experimental study in Bihar, India. PLoS ONE. 2017;12:e0171002. 28146586 10.1371/journal.pone.0171002 PMC5287473

[19] Iyengar K. Early postpartum maternal morbidity among rural women of Rajasthan, India: a community-based study. J Health Popul Nutr. 2012;30:213–25. 22838163 10.3329/jhpn.v30i2.11316 PMC3397332

[20] Nithya R, Dorairajan G, Chinnakali P. Do pregnant women know about danger signs of pregnancy and childbirth? – A study of the level of knowledge and its associated factors from a tertiary care hospital in Southern India. Int J Adv Med Health Res. 2017;4:11.

[21] Ozbay F, Johnson DC, Dimoulas E, Morgan CA, Charney D, Southwick S. Social support and resilience to stress: from neurobiology to clinical practice. Psychiatry (Edgmont). 2007;4:35–40. 20806028 PMC2921311

[22] Haleema M, Raghuveer P, Kiran R, Mohammed IM, Mohammed ISA, Mohammed M. Assessment of knowledge of obstetric danger signs among pregnant women attending a teaching hospital. J Family Med Prim Care. 2019;8:1422–6. 31143733 10.4103/jfmpc.jfmpc_149_19 PMC6510108

[23] Aruldas K, Kant A, Mohanan PS. Care-seeking behaviors for maternal and newborn illnesses among self-help group households in Uttar Pradesh, India. J Health Popul Nutr. 2017;36(S1):49. 29297413 10.1186/s41043-017-0121-1 PMC5764050

[24] Legare CH, Akhauri S, Chaudhuri I, et al. Perinatal risk and the cultural ecology of health in Bihar, India. Philos Trans R Soc Lond B Biol Sci. 2020;375:20190433. 32594881 10.1098/rstb.2019.0433 PMC7423251

[25] Bedaso A, Adams J, Peng W, Sibbritt D. The relationship between social support and mental health problems during pregnancy: a systematic review and meta-analysis. Reprod Health. 2021;18:162. 34321040 10.1186/s12978-021-01209-5 PMC8320195

[26] Ranjan Kumar P, Unisa S. Effect of social support networks on maternal knowledge of child health in rural Odisha, India. J Health Soc Sci. 2017;2:99–118.

[27] Upadhyay RP, Chowdhury R, Aslyeh S, et al. Postpartum depression in India: a systematic review and meta-analysis. Bull World Health Organ. 2017;95:706-717C. 29147043 10.2471/BLT.17.192237 PMC5689195

[28] El Ayadi A, Nalubwama H, Barageine J, et al. Development and preliminary validation of a post-fistula repair reintegration instrument among Ugandan women. Reprod Health. 2017;14:109. 28865473 10.1186/s12978-017-0372-8 PMC5581461

[29] International Institute for Population Sciences (IIPS) and ICF. National Family Health Survey (NFHS-5), 2019–21. Mumbai: International Institute for Population Sciences; 2021, http://rchiips.org/nfhs/NFHS-5Reports/NFHS-5_INDIA_REPORT.pdf. Accessed 5 May 2022.

[30] Kumar P, Srivastava S, Maurya C, Dhillon P. An assessment of the role of socio-economic, maternal and service utilization factors in increasing self-reported maternal complications in India. BMC Pregnancy Childbirth. 2021;21:519. 34289804 10.1186/s12884-021-03997-x PMC8296634

[31] Saunders B, Sim J, Kingstone T, et al. Saturation in qualitative research: exploring its conceptualization and operationalization. Qual Quant. 2017;52:1893. 29937585 10.1007/s11135-017-0574-8 PMC5993836

[32] Berkman L, Glass T. Social integration, social networks, social support, and health. In: Social epidemiology. New York: Oxford University Press; 2000.

[33] Panda S, D’Sa J, Rao AC. Support needs of Indian women in early labour. JWRH. 2016;1:15–24.

[34] Yadav D, Dabas K, Malik P, Bhandari A, Singh P. “Should I visit the clinic”: analyzing WhatsApp-mediated online health support for expectant and new mothers in Rural India. In: Proceedings of the 2022 CHI Conference on Human Factors in Computing Systems. New York: Association for Computing Machinery; 2022. p. 1–20.

[35] Al-Mutawtah M, Campbell E, Kubis H-P, Erjavec M. Women’s experiences of social support during pregnancy: a qualitative systematic review. BMC Pregnancy Childbirth. 2023;23:782. 37950165 10.1186/s12884-023-06089-0 PMC10638802

[36] Felisian S, Mushy SE, Tarimo EAM, Kibusi SM. Sociocultural practices and beliefs during pregnancy, childbirth, and postpartum among indigenous pastoralist women of reproductive age in Manyara, Tanzania: a descriptive qualitative study. BMC Womens Health. 2023;23:123. 36959588 10.1186/s12905-023-02277-4 PMC10035110

[37] Omer S, Zakar R, Zakar MZ, Fischer F. The influence of social and cultural practices on maternal mortality: a qualitative study from South Punjab, Pakistan. Reprod Health. 2021;18:97. 34006307 10.1186/s12978-021-01151-6 PMC8130310

[38] Sumankuuro J, Crockett J, Wang S. Sociocultural barriers to maternity services delivery: a qualitative meta-synthesis of the literature. Public Health. 2018;157:77–85. 29501985 10.1016/j.puhe.2018.01.014

[39] International Institute for Population Sciences. National family health survey-4 2015–2016. New Delhi: Government of India, Ministry of Health and Family Welfare; 2017.

[40] What he knows about her and how it affects her? Husband’s knowledge of pregnancy complications and maternal health care utilization among tribal population in Maharashtra, India | BMC Pregnancy and Childbirth | Full Text. https://bmcpregnancychildbirth.biomedcentral.com/articles/10.1186/s12884-019-2214-x. Accessed 23 Apr 2025. 10.1186/s12884-019-2214-x PMC6373054 30760234

[41] Emagneneh T, Mulugeta C, Ejigu B, et al. Men’s knowledge of obstetrics danger sign and associated factors in low-income countries: a systematic review and meta-analysis. Sci Rep. 2025;15:6560. 39994327 10.1038/s41598-025-89541-9 PMC11850788

[42] Kaushal P, Khapre M, Das A, Kumari R, Sharma M. Community perspective of male involvement in maternal health care in Uttarakhand, India: a qualitative study. J Obstet Gynaecol India. 2022;73:113. 37073237 10.1007/s13224-022-01672-5 PMC10105805

[43] Chayal V, Sagar V, Verma R, et al. Husband’s involvement in utilization of maternal health services by their spouse in district Rohtak, Haryana. J Family Med Prim Care. 2024;13:2272. 39027839 10.4103/jfmpc.jfmpc_1153_23 PMC11254083

[44] Varghese S, Singh S, Kour G, Dhar T. Knowledge, attitude and preferences of pregnant women towards mode of delivery in a tertiary care center. Int J Res Med Sci. 2016;4:4394–8.

[45] Ahmad D, Mohanty I, Hazra A, Niyonsenga T. The knowledge of danger signs of obstetric complications among women in rural India: evaluating an integrated microfinance and health literacy program. BMC Pregnancy Childbirth. 2021;21:79. 33485310 10.1186/s12884-021-03563-5 PMC7824939

[46] Macones GA, Caughey AB, Wood SL, et al. Guidelines for postoperative care in cesarean delivery: enhanced recovery after surgery (ERAS) Society recommendations (part 3). Am J Obstet Gynecol. 2019;221:247.e1-247.e9. 30995461 10.1016/j.ajog.2019.04.012

[47] Allendorf K. The quality of family relationships and use of maternal health-care services in India. Stud Fam Plann. 2010;41:263–76. 21465727 10.1111/j.1728-4465.2010.00252.x PMC4845731

[48] Gayesa RT, Ngai FW, Xie YJ. The effects of mHealth interventions on improving institutional delivery and uptake of postnatal care services in low-and lower-middle-income countries: a systematic review and meta-analysis. BMC Health Serv Res. 2023;23:611. 37296420 10.1186/s12913-023-09581-7 PMC10257264

[49] Lee SH, Nurmatov UB, Nwaru BI, Mukherjee M, Grant L, Pagliari C. Effectiveness of mHealth interventions for maternal, newborn and child health in low- and middle-income countries: systematic review and meta-analysis. J Glob Health. 2016;6:010401. 26649177 10.7189/jogh.06.010401 PMC4643860

[50] Qian J, Wu T, Lv M, et al. The value of mobile health in improving breastfeeding outcomes among perinatal or postpartum women: systematic review and meta-analysis of randomized controlled trials. JMIR mHealth Uhealth. 2021;9:e26098. 34269681 10.2196/26098 PMC8325083

[51] Sakamoto JL, Carandang RR, Kharel M, et al. Effects of mHealth on the psychosocial health of pregnant women and mothers: a systematic review. BMJ Open. 2022;12:e056807. 35168981 10.1136/bmjopen-2021-056807 PMC8852716

[52] Sondaal SFV, Browne JL, Amoakoh-Coleman M, et al. Assessing the effect of mHealth interventions in Improving maternal and neonatal care in low- and middle-income countries: a systematic review. PLoS ONE. 2016;11:e0154664. 27144393 10.1371/journal.pone.0154664 PMC4856298

